# A Step Backward

**Published:** 1903-09

**Authors:** 


### A STEP BACKWARD.

The National Association of Dental Examiners, at its recent meeting at Asheville, N. C., made a radical change in its methods of dealing with the dental colleges of the United States. Heretofore this national body had a list prepared yearly of so-called accepted dental colleges. This was made up of the colleges in membership with the Association of Faculties. This was supposed to be a guide for the State boards, and was so recognized by them. Hence the graduates of those dental colleges not upon the list were refused recognition outside of the State in which the college was situated. Several instances are on record where colleges attempted to act independently of this organization and the "Faculties," with an invariably disastrous result. So universally has this resulted that every effort has been made to be taken back in the Association of Faculties, which meant acceptance by the "Examiners" and a relisting of the college.

Now all this has been changed, if the writer understands the recent action of this body. The applicant for license from State boards may come from a college having three years, four years, or no years at all, and be examined, where the laws of the States will permit it. The National Association of Dental Examiners simply demands that he shall make a certain advanced per cent. in his examination.

If this is not going back to primitive methods, then the writer has misunderstood the drift of this action and stands ready to be corrected. It repudiates, in effect, at least, all college education, and is based on the Massachusetts State law, which declares: "Sec. 4. All persons who shall have attained the age of twenty-one years . . . may appear before said board at any of its regular meetings and be examined with reference to their knowledge and skill in dentistry and dental surgery, etc." If satisfactory, the certificate is issued regardless of a diploma. This may be all well in theory, and in isolated instances may be correct in practice, but if followed out to its logical conclusion it would do away with all college and school work, for instances are constantly being recorded of self-education, so-called, in all lines of work. It is not believed, however, that there was ever an individual, thus selfeducated, that did not regret to the day of his death that he had failed to have had the experience of college training.

The writer is not pessimistic and ready to believe that the effect of this action of the Association of Examiners will, in the end, destroy dental colleges. These will live long after the National and State boards have been forgotten, but the discouraging effect cannot be minimized.

The National Association of Examiners is congratulating itself that its membership has taken an advanced step. It is believed by the writer that this means, in theory, at least, a relapse into the methods in vogue before the Association of Faculties was organized. It, in fact, nullifies the good that the Association hoped to accomplish as the result of the four years' course. Indeed, it is questionable whether under this ruling a college could not continue the present three years'. It would be liable to expulsion from the organization, but that would not materially affect its interests so long as it could bring its students up to the per cent. demanded.

For years the State boards and National Association of Dental Examiners have assumed that it has been through their combined efforts that dentistry has been forced to advance to its present high position. The writer has invariably asserted that this claim was not founded upon any historical evidence; that the advance has been a necessary evolution and made by the colleges, and would have taken place sooner or later in the natural order of progress without their aid. Now, when dental education sinks to a lower level in this country, the legitimate result of this 1903 action, they will probably charge the retrograde to the colleges and not to their unwise action. A step forward is always in order, but a step backward is sure to result in disaster, and this latter the National Association of Dental Examiners has taken. It is to be hoped that the dental colleges are so firmly based on the best educational foundation that they will not for a moment listen to the indirect temptation held out to them by this body, but will advance, firmly united, regardless of paper resolutions of boards, State or national.

